# Separating fetal and maternal placenta circulations using multiparametric MRI

**DOI:** 10.1002/mrm.27406

**Published:** 2018-09-21

**Authors:** Andrew Melbourne, Rosalind Aughwane, Magdalena Sokolska, David Owen, Giles Kendall, Dimitra Flouri, Alan Bainbridge, David Atkinson, Jan Deprest, Tom Vercauteren, Anna David, Sebastien Ourselin

**Affiliations:** ^1^ Department of Medical Physics and Biomedical Engineering University College London London United Kingdom; ^2^ School of Biomedical Engineering and Imaging Kings College London, London United Kingdom; ^3^ Institute for Women’s Health University College Hospital,London London United Kingdom; ^4^ Medical Physics University College Hospital London United Kingdom; ^5^ Centre for Medical Imaging University College London London United Kingdom; ^6^ University Hospital KU Leuven Leuven Belgium; ^7^ NIHR University College London Hospitals Biomedical Research Centre London United Kingdom

**Keywords:** chorion, DECIDE, diffusion, flow‐matching, relaxometry

## Abstract

**Purpose:**

The placenta is a vital organ for the exchange of oxygen, nutrients, and waste products between fetus and mother. The placenta may suffer from several pathologies, which affect this fetal‐maternal exchange, thus the flow properties of the placenta are of interest in determining the course of pregnancy. In this work, we propose a new multiparametric model for placental tissue signal in MRI.

**Methods:**

We describe a method that separates fetal and maternal flow characteristics of the placenta using a 3‐compartment model comprising fast and slowly circulating fluid pools, and a tissue pool is fitted to overlapping multiecho T2 relaxometry and diffusion MRI with low b‐values. We implemented the combined model and acquisition on a standard 1.5 Tesla clinical system with acquisition taking less than 20 minutes.

**Results:**

We apply this combined acquisition in 6 control singleton placentas. Mean myometrial T2 relaxation time was 123.63 (±6.71) ms. Mean T2 relaxation time of maternal blood was 202.17 (±92.98) ms. In the placenta, mean T2 relaxation time of the fetal blood component was 144.89 (±54.42) ms. Mean ratio of maternal to fetal blood volume was 1.16 (±0.6), and mean fetal blood saturation was 72.93 (±20.11)% across all 6 cases.

**Conclusion:**

The novel acquisition in this work allows the measurement of histologically relevant physical parameters, such as the relative proportions of vascular spaces. In the placenta, this may help us to better understand the physiological properties of the tissue in disease.

## 1 INTRODUCTION

The placenta is uniquely interesting as a biological tissue facilitating the exchange of nutrients and waste products between 2 or more individuals.^1^ The efficiency of the placenta thus depends on the interaction of independent circulatory systems and a tissue substrate allowing close, but nonmixing, blood pools. Problems associated with this circulation and exchange may be detrimental to both the mother and fetus and include both early‐ and late‐onset fetal growth restriction,^2^ complicated twins,^3^, ^4^ and pre‐eclampsia,^5^ with high associated levels of morbidity and mortality for both. Several of these conditions lead to observable histological differences in the postpartum placenta.^6^, ^7^

Monitoring placental function using MRI is a growing research area,^8^ but in comparison to neonatal imaging, multicontrast studies are almost entirely absent from the literature owing to the unpredictable nature and time constraints of fetal and placental MRI. Despite this, several contrasts from MRI have been investigated for monitoring placental blood flow and function, each with their own advantages and disadvantages.^9^, ^10^ Dynamic contrast‐enhanced (DCE) MRI is the gold standard for measurement of whole‐organ vascular function in MRI. The technique has been used extensively in the primate placenta,^11^, ^12^ and the relative similarity of the invasive placenta structure makes this a relevant animal model. Animal models such as these enable the comparison of markers of vascular function obtained from multiple MRI modalities, allowing us to better understand and enhance the more limited information that can be obtained in human placental imaging, for instance by obtaining precise markers of pharmacokinetic function alongside markers of oxygenation status.^11^ Because of the use of exogenous contrast, which is cautiously used in nonpregnant adults, DCE‐MRI use is unlikely to become widespread because of the associated limitations in our understanding of how the contrast agent is cleared from the feto‐maternal system.^13^ Despite this, certain pregnancy conditions exist in which human DCE‐MRI would be carried out, and it represents the benchmark against which future studies for placental blood flow and physiology will need to be compared.^9^ As an endogenous contrast mechanism, arterial spin labeled (ASL) MRI also holds much promise for understanding feto‐placental blood delivery, but is currently limited by low signal‐to‐noise ratio (SNR) and exquisite susceptibility to motion.^14^, ^15^

Of the techniques without the use of exogenous contrast agent, diffusion‐weighted imaging (DWI) is becoming increasingly widespread in abdominal and placental imaging and is widely used in the body in general, especially in the liver.^16^, ^17^, ^18^, ^19^ When combined with the intravoxel incoherent motion model (IVIM) of blood flow in capillaries, it provides a noninvasive method of measuring tissue properties relating to flow and perfusion. IVIM has been measured in the placenta of small for gestational age (SGA) pregnancies and found to correlate well with the current clinical measure of maternal placental perfusion (uterine artery Doppler ultrasound).^18^ The IVIM model can also be adapted for the placenta to make it sensitive to the high net directionality of flow in the chorionic and stem vessels, and there is some evidence now that this directionality can be measured by IVIM.^19^ T2 relaxometry, made possible by the acquisition of images with variable echo time, provides additional information on the static tissue composition and intrinsic tissue T2 value. T2 relaxometry is particularly interesting because of the quite large effect size observed in studies of SGA fetuses and its relationship to blood oxygenation observed in primate and human populations.^11^, ^21^, ^22^, ^23^ It is thus an obvious candidate for assessing placental function in a range of other placental conditions.^24^ Extensive recent work has also been carried out investigating T1 relaxometry^20^, ^25^, ^26^, ^27^ and T2* relaxometry, which is intrinsically linked to blood oxygenation.^11^, ^21^, ^22^, ^23^ Notably, there is a high spatial variability in placental T2* maps that has been observed,^11^ which corresponds to the known macroscopic lobular structure of the placenta and its division into discrete functional units. In this sense, the placenta can be spatially compartmentalized by observed function from multiple MRI contrasts such as DCE‐MRI or T2*. Here, we discuss this compartmentalization within the level of the individual voxel, below the spatial resolution of MRI.

The structure of the placenta is such that even within a voxel, there are likely to be 2 distinct flow compartments from the mother and baby in addition to the placental tissue. Deep within the placenta, the fetal blood is constrained to the capillaries and circulates rapidly, driven by the rapid heart rate of the fetus. As a result, this meets the assumptions of the IVIM model, provided there is no directional preference in fluid flow at this scale. On the chorionic plate, this assumption does not hold given that the chorionic and stem vessels receive relatively high pulsatile flow from the umbilical arteries. This flow is directional and has a flow profile different from the returning flow in the chorionic veins and, as a result, has been shown to be measurable on diffusion weighted MRI.^19^ Fortunately, this directionality in the chorionic and stem vessels is constrained by the shape of the in utero chorionic plate, making this a well‐defined geometric feature of the fetoplacental vasculature. Between the fetal and maternal circulations, there exists a trophoblastic space representing the space through which gases and nutrients diffuse or are transported. Conceptually, this is a tissue‐dense space and water in this space is, broadly speaking, undergoing physically hindered Brownian motion. The third major component of the placenta is the maternal fluid compartment. Blood is injected by the transformed spiral arteries into the placenta, which, at late gestation, will ordinarily be a low‐velocity, high‐volume configuration, attributed to remodeling in early pregnancy. Given that the velocity is much lower than that in the fetal capillaries,^6^, ^28^ this will appear to a first approximation as a large fluid‐filled space with correspondingly long T2 and diffusivity close to that of pure water at body temperature. The distinct compartments of the fetal capillaries, trophoblast space, and maternal blood pool thus have separate MR properties of both diffusivity and relaxivity. By applying a multicomponent model, we intend to disentangle the signal from each of the compartments and begin to understand how the placenta is functioning and how the respective fetal and maternal circulations are physiologically matched.

The effects observed using both IVIM and T2 relaxometry are dependent upon one another and how they are acquired. With separate studies measuring only 1 parameter, it will be difficult to isolate changes to the structural T2 measurement from the functional flow measurement of IVIM. To address this problem, we develop a multicontrast, multicompartment MRI‐based model of the placenta consisting of a 3‐compartment model that combines overlapping variable diffusion weighting and echo time to measure markers of underlying microvascular properties in the placenta.^29^ We term this joint placenta model and acquisition Direct parameter Estimation from Combined Imaging Data (Diffusion‐rElaxation Combined Imaging for Detailed Placental Evaluation; DECIDE). We show that this new joint acquisition and modeling can produce measurements that reflect the underlying contributions of fetal and maternal circulations at the voxel‐wise level; this technique may thus be relevant for a number of placental pathologies. Additionally, we propose 2 new functional biomarkers derived from this imaging data:

- the maternal‐fetal blood volume ratio, which examines matching of fetal and maternal blood throughout the placenta, on a voxel‐wise basis.
- the fetal blood saturation measured throughout the placenta, presenting values for the mean placental fetal blood oxygen saturation.

## 2 METHODS

### 2.1 Data

The study was approved by the local research ethics committee, and all subjects gave written informed consent (London–Hampstead Research Ethics Committee, REC reference 15/LO/1488). We acquired data from 6 uncomplicated singleton placentae at (29 + 1, 31 + 3, 28 + 4, 34 + 0, 28 + 5, and 25 + 1) gestational weeks (+days) for cases (1, 2, 3, 4, 5, and 6) respectively. Inclusion criteria were 24 to 34 weeks’ gestational age with normal anomaly ultrasound scan, estimated fetal weight greater than the 10th centile, amniotic fluid deepest pool measurement in the normal range for gestation and normal umbilical artery Doppler measurements on ultrasound scan done within 1 week of the MRI scan, and no known maternal complications (no evidence of pre‐eclampsia, hypertension, or low risk for preterm labor).

Imaging was performed under free breathing on 1.5 Tesla (T) Siemens Avanto (Siemens, Erlangen, Germany), at 7 b‐values (**b**; 0, 50, 100, 150, 200, 400, and 600 $s.mm^{-2}$) and 10 echo times (**T_E_**; 81, 90, 96, 120, 150, 180, 210, 240, 270, and 300 ms). All echo times were acquired at b‐value = 0 $s.{\mathrm{mm}}^{-2}$, to allow T2 fitting, and all b‐values at **T_E _**= 96ms. In addition, data were acquired at b‐value 50 and 200$s.mm^{-2}$ for **T_E _**= (81, 90, 120, 150, 180, 210, and 240) ms, to allow simultaneous sampling of diffusivity and relaxivity (see Table 1 for more detail). Voxel resolution was 1.9 × 1.9 × 6 mm. Total acquisition time was 20 minutes. Variable repetition time (TR) was used to reduce imaging time. All images were acquired with the same echo‐planar readout, and the TR was held at 3900ms, but allowed to vary up to 9200ms for longer echo‐time measurements. Because of typical abdominal T1 values, differences in signal attributed to variable TR are expected to be small and the effect of otherwise lengthening the scan using fixed TR will reduce its clinical utility.

**Table 1:** Image acquisition parameters for DECIDE

		Echo time (ms)										
b‐value (s.mm^–2^)	0	96	81	90	120	150	180	210	240	270	300	
	50	96		90	120	150	180					
	100	96										
	150	96										
	200	96	81	90	120	150	180	210	240			
	400	96										
	600	96										
	^*^41 measurements total (images at 96 ms TE acquired 3 times).											

Images at 96 ms echo time (TE) constitute an IVIM‐like acquisition and are acquired 3 times across slice‐phase‐read directions.

To minimize the effect of motion, we first used a nonrigid registration routine to align all images and then manually segmented the placenta.^30^, ^31^ Masks were drawn manually over the area of interest in the registered multiple slices of the 2D stack (itk‐SNAP Version 3.2.0, 2014). The placenta and a section of retroplacental myometrium were segmented. Segmentations were drawn fully within the boundary of the placenta to minimize noise from movement entering the analysis.

### 2.2 DECIDE: multicompartment placenta modeling

The DECIDE model is a multicompartment model of placental perfusion that combines T2 relaxometry and DWI.^29^ Intracapillary fetal blood has high pseudo‐diffusivity, $d^{*}$, and long T2 relaxation time, $T_{2}^{\mathrm{fb}}=1/R_{2}^{\mathrm{fb}}$ and volume fraction f. Maternal blood with volume fraction, ν, is in the intervillous space, as opposed to intravascular, and therefore has lower diffusivity, d, and slow relaxation, $R_{2}^{\mathrm{mb}}$. Finally, the remaining signal from the tissue has low diffusivity, d, and rapid relaxation, $R_{2}^{\mathrm{ts}}$, associated with dense tissue.^16^, ^25^, ^32^

### 2.3 Extending the DECIDE model to fit T2 relaxation times

Making use of T2 relaxation values from the literature, rather than measured T2 values, may add bias to the model, given that both maternal and fetal blood characteristics are different from the normal, healthy adult.^29^ It is normal for pregnant women to have a physiological anemia and therefore reduced hematocrit. This may affect the T2 relaxation time of blood, which is known to be sensitive to hematocrit and oxygen saturation. Fetal blood has higher hematocrit and lower oxygen saturation than adult blood. Deoxygenated fetal blood in the umbilical artery (supplying the placenta) at 30 weeks gestational age is estimated to be 65% saturated, and oxygenated blood returning to the fetus in the umbilical vein is estimated to be 85% saturated.^33^ This is much lower than normal adult saturations of 97%to100%. We adapted the DECIDE model to compare typical T2 values of maternal and fetal blood.^34^

### 2.4 Model‐fitting routines

All model fitting was done using in‐house software developed in MATLAB (The MathWorks Inc., Natick, MA). We developed a bespoke fitting routine for these data to improve model‐fitting performance with 2 key features.

#### 2.4.1 Region of interest parameter initialization

In the presence of noisy data, nonlinear models with several free parameters can be prone to fitting to local minima. We avoided this situation by making use of model‐fitting results obtained from average region of interest (ROI) signal curves. This has the effect of boosting the SNR, yielding robust ROI parameter estimates from the average signal curve, which make reasonable starting estimates for fitting at the voxel level within the ROI. We thus initialized our nonlinear fitting routines with parameter estimates from larger placental and myometrial ROIs.

#### 2.4.2 Fitting of independent parameters

Models often contain parameters that are dependent only weakly on some parts of the underlying data. A good example of this is in diffusion MRI, where apparent diffusion coefficient (ADC) measurements can be robustly obtained from high b‐value monoexponential data and the ADC is only weakly dependent on low b‐value effects.^16^ Prefitting independent parameters is a common approach for IVIM model fitting, and we made use of this constraint when fitting our data. We also made use of this technique when applying the standard IVIM model to both the myometrial and placenta data sets.

#### 2.4.3 Myometrium model fitting

The myometrium is a maternally perfused, highly vascular, muscular tissue and is not expected to have a significant pooled‐fluid compartment. Thus, it is reasonable to assume a modified DECIDE model, with 2 compartments representing an IVIM blood pool at high oxygen saturation and a dense tissue space of much lower T2 (Equation ((1))).^16^

(1) $$S\left(b,t\right)=S_{0}\left[{\mathrm{fe}}^{-bd^{*}-T_{E}R_{2}^{\mathrm{mb}}}+\left(1-f\right)e^{-bd-T_{E}R_{2}^{\mathrm{ts}}}\right]$$

1. *ADC* d *fitting:* We applied log‐linear fitting of both whole‐ROI and voxel‐wise d values to the data with b‐value b>100 for fixed echo time.
2. *Estimation of myometrial maternal blood T2:* Equation ((1)) is fitted to the high‐SNR average signal curve from the whole myometrial ROI. Average whole‐placenta estimates of f, $d^{*}$, $R_{2}^{\mathrm{mb}}$, and $R_{2}^{\mathrm{ts}}$ are obtained. ADC, d, is constrained as in step 1.
3. *Estimation of myometrial volume fractions:* For each voxel, we fix the local value of the ADC (step 1) and obtain nonlinear fits of f, $d^{*}$, $R_{2}^{\mathrm{mb}}$, and $R_{2}^{\mathrm{ts}}$, initialized with the global tissue estimates from step 2.

#### 2.4.4 Placenta model fitting

We apply the DECIDE model to fit placental tissue (Equation ((2))) with variables as defined above.

(2) $$\begin{array}{c} S\left(b,\;t\right)=S_{0}[fe^{-bd^{*}-T_{E}R_{2}^{\mathrm{fb}}}+\left(1-f\right)e^{-bd}\\ (\nu e^{-T_{E}^{}R_{2}^{\mathrm{mb}}}+\left(1-\nu \right)e^{-T_{E}R_{2}^{\mathrm{ts}}})] \end{array}$$

We apply the same fitting approach described as for the myometrium, modifying step 2 accordingly so that we may fit fetal blood relaxation $R_{2}^{\mathrm{fb}}$, $R_{2}^{\mathrm{mb}}$, and $R_{2}^{\mathrm{ts}}$ are held fixed at literature values of ${\left(240\mathrm{ms}\right)}^{-1}$ and ${\left(46\mathrm{ms}\right)}^{-1}$, respectively, whereas all other parameters are fitted.^25^, ^32^, ^34^

#### 2.4.5 Estimation of in utero fetal blood oxygen saturation

Fitting an empirical curve to the data enables us to estimate oxygen saturation values for known T2 (Equation ((3))), based on data from a previous work.^34^

(3) $$T2\left(s\right)=a/\left(1+e^{-g(s-c)}\right)$$

Fitted parameters for this curve, given fractional saturation, s, are *a = 386 *ms, *g = 0.36*, and *c = 0.88*. We are thus able to find approximate saturation values for each known T2 blood pool.

## 3 RESULTS

### 3.1 Whole placenta curve fitting

**Figure 1:**
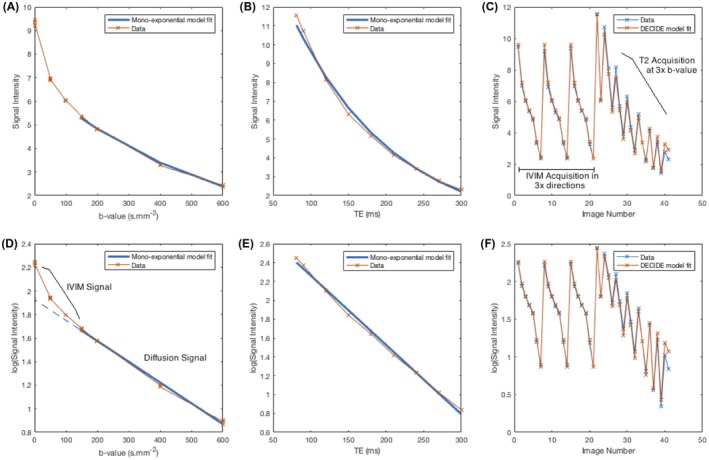
Whole‐placenta curve fitting results for fixed echo‐time IVIM imaging (case 2) for signal (A) and log‐signal (D) (curve fitting for b > 100 s.mm^–2^). For zero b‐value, monoexponential relaxometry for signal (B) and log‐signal (E). For DECIDE fitting (Equation ((3))), a single model‐fitting procedure to all acquired images for signal (C) and log‐signal (F)

Figure 1 shows the results of model fitting to the average signal from 1 of the control placentas (control case 2). Figure 1A shows the diffusion‐weighted signal curve for fixed T_E_ and variable b‐value. The curve is highly biexponential at low b‐values, shown by the extrapolated linear fit in the equivalent log‐transformed signal shown in Figure 1D, which should be a straight‐line relationship for a monoexponential function. The fitted line in this example is for linear fitting to b‐values greater than 100$s.mm^{-2}$. Comparably, the results of monoexponential fitting for variable echo time and fixed b‐value = 0$s.mm^{-2}$ are shown in Figure 1B (signal) and 1e (log‐signal). For variable echo time, evidence of multiexponential behavior is more subtle and represented as a slight bias in the curve shape relative to the fitted curve. This is likely related to the, on average, relatively low volume of short T2 cell‐dense space relative to the volume of blood in both maternal and fetal circulations, which have much higher diffusivity and much longer echo time.^16^, ^32^ Figure 1C and 1f shows the MR signal and DECIDE model fit (and log signal) obtained for the 41 images. Imaging is obtained by acquiring 3 IVIM experiments consecutively with different diffusion‐encoding directions, followed by progressively lengthening echo‐time data with variable diffusion weighting. Overlaid on these plots is the result of a DECIDE multicompartment fit showing close matching of the fitted data to the original signal curve.

### 3.2 Myometrium parametric maps

**Figure 2:**
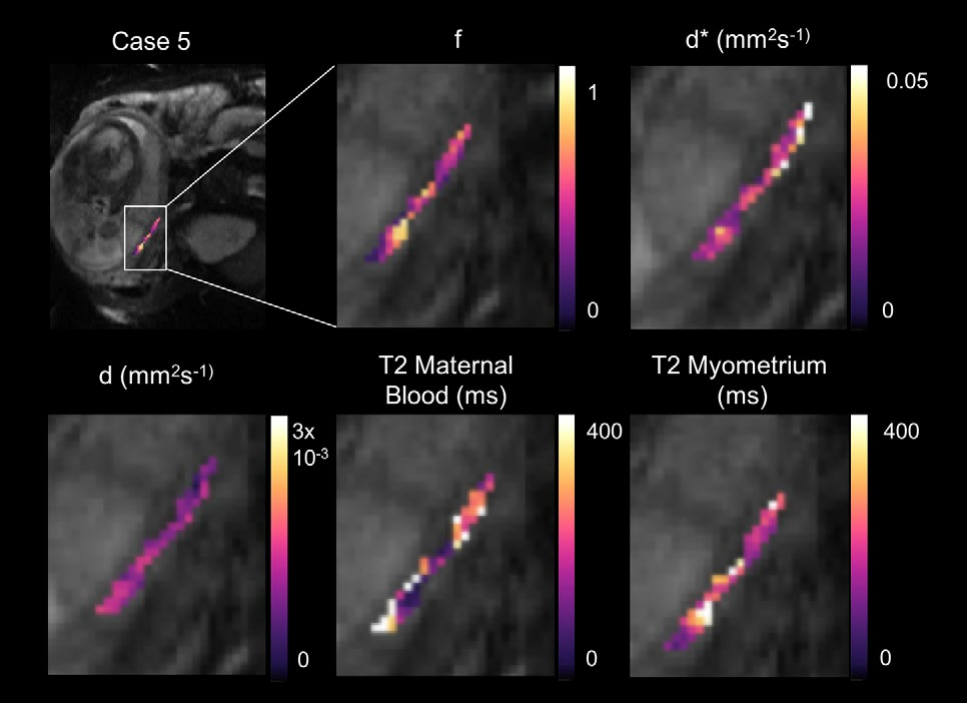
Example parametric maps for the T2‐IVIM fit for 1 case where myometrial analysis was feasible. Panels show parameter maps, from left to right, f, d*, d, T2 maternal blood, and T2 myometrium. Example figures for all cases are shown in the Supporting Information

Myometrial parameter maps for 1 case (2 were rejected for having significant motion in this region) is shown in Figure 2 (parametric maps for all subjects are shown in Supporting Information Figure S1). f was high in the myometrium, which is expected given that this is a highly vascular tissue in pregnancy, delivering maternal blood to the placenta by the remodeled, dilated spiral arteries.

**Figure 3:**
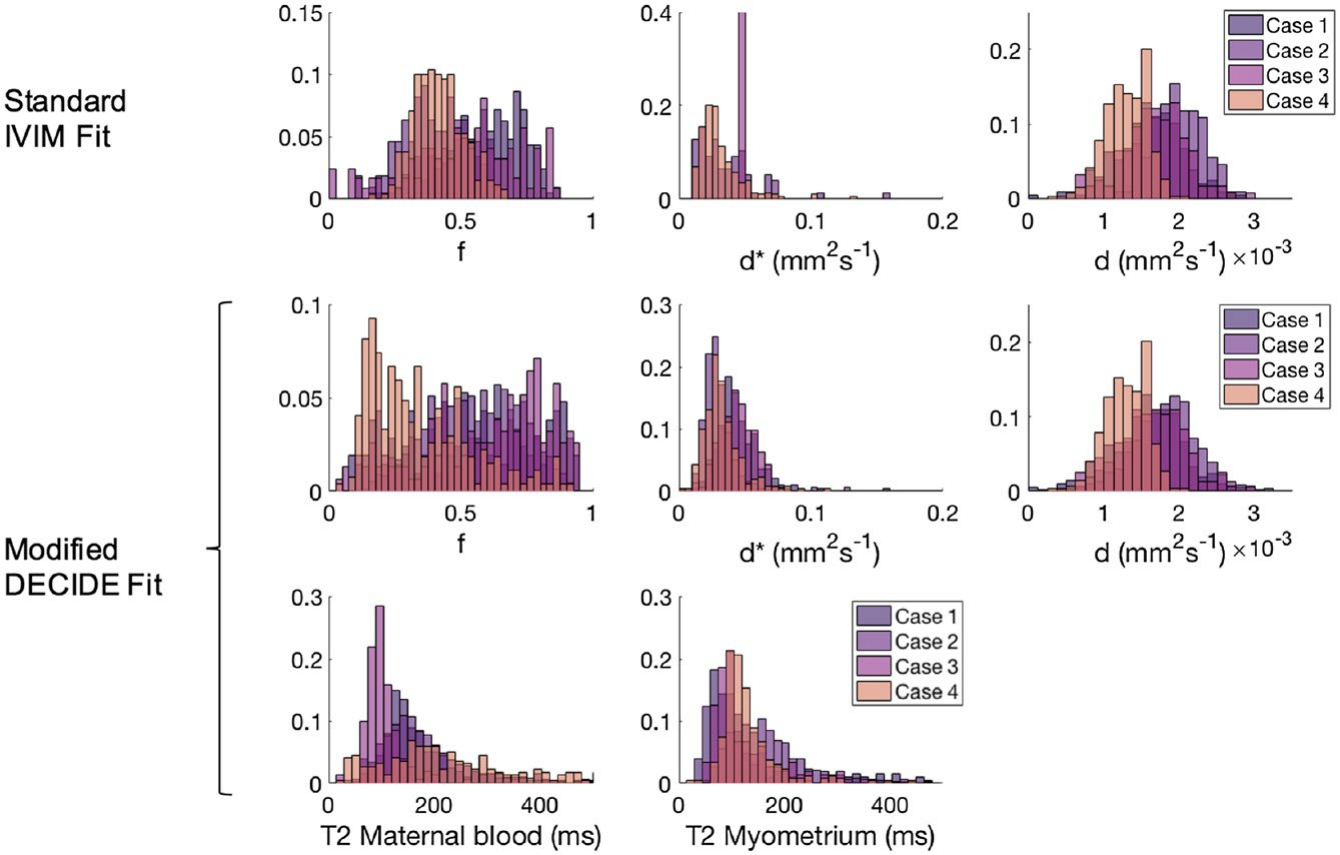
Histograms for the voxel‐wise fit, for the 4 myometrial data sets. Row 1 shows f, d*, and d for the standard IVIM fit. Rows 2 and 3 show f, d*, d, and T2 relaxation time of maternal blood and myometrium for the combined myometrium fit

Figure 3 shows histograms of the fit for each myometrial case for each parameter, using the standard IVIM model and the myometrial model in Equation ((1)). Table 2 shows a comparison of the mean (±standard deviation) for each parameter for the standard IVIM fit and the modified DECIDE fit for the myometrium. Estimates for f and d* were found to be comparable between the 2 fits.^16^ Mean myometrial T2 relaxation time was 123.63 (±6.71) ms.^32^ Mean T2 relaxation time of maternal blood was 202.17 (±92.98) ms. These results obtained for the T2 relaxation time of maternal blood are not significantly different from 240 ms.^34^ Based upon current knowledge, it is difficult to justify why values of T2 would differ systematically in the presence of relatively indistinguishable hematocrit and saturation; thus, this motivated us to fix this value across the participants at a value of 240 ms throughout the subsequent placenta model fitting (Equation ((2))).

**Table 2:** Mean and standard deviation (STD) of voxel‐wise fit for the myometrial and placenta fit

**Myometrium**			**Placenta**		
	**Standard IVIM** **fit mean (±STD)**	**T2 IVIM** **fit mean (±STD)**		**Standard IVIM** **fit mean (±STD)**	**DECIDE fit mean (±STD)**
f	0.48 (0.087)	0.44 (0.14)	f	0.27 (0.026)	0.23 (0.034)
d* / mm^2^s^–1^	0.051 (0.013)	0.044 (0.008)	d* / mm^2^s^–1^	0.034 (0.003)	0.028 (0.005)
d / mm^2^s^–1^	0.0016 (0.0003)	0.0016 (0.0003)	d / mm^2^s^–1^	0.0017 (0.0001)	0.0017 (0.0001)
T2 maternal blood / ms		202.17 (92.98)	v		0.318 (0.062)
T2 myometrium / ms		123.63 (6.71)	T2 fetal blood / ms		144.89 (54.42)

Left panel: Myometrial fit for f and d* using the standard IVIM and the myometrial model (Equation ((1))) and T2 relaxation time of maternal blood and myometrium using the myometrial model. T2 relaxation times are consistent with values previously reported in the literature.^21^, ^22^ Right panel: placental fit for f and d* using the standard IVIM and the DECIDE model (Equation ((2))), and and T2 relaxation time of fetal blood with the DECIDE model. The placenta shows a significant third compartment (v). Fetal blood T2 relaxation time is lower than the literature value for adult blood T2.^21^

### 3.3 Placenta parametric maps

**Figure 4:**
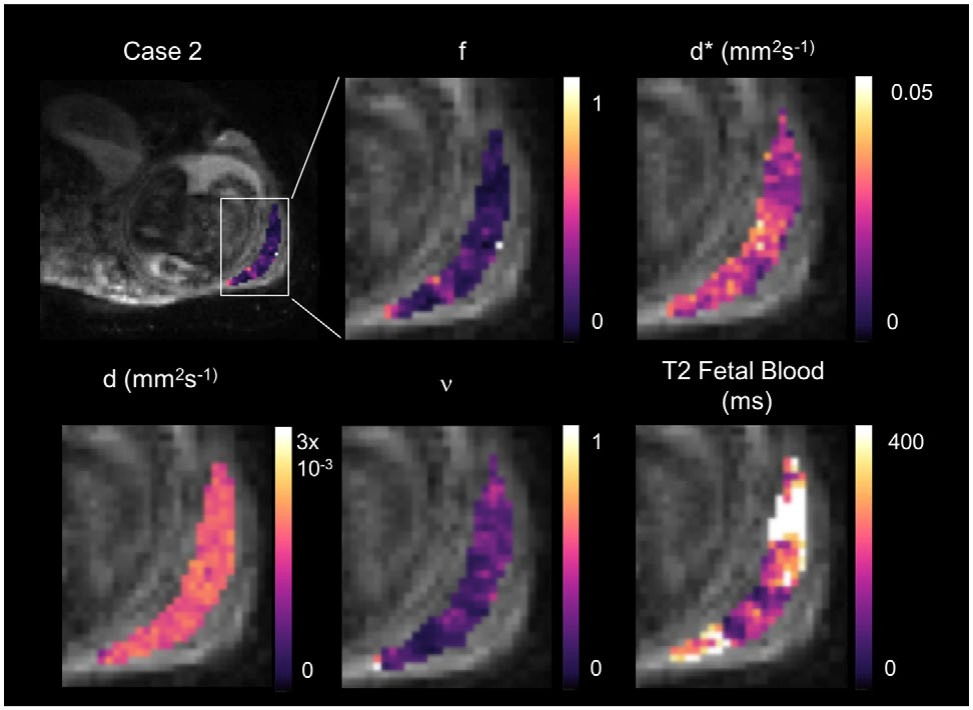
Parametric maps for the DECIDE fit (Equation ((2))) for 1 control singleton pregnancy. Panels show parameter maps, from left to right, f, d*, d, v, and T2 fetal blood. Example figures for all cases are shown in the Supporting Information

**Figure 5:**
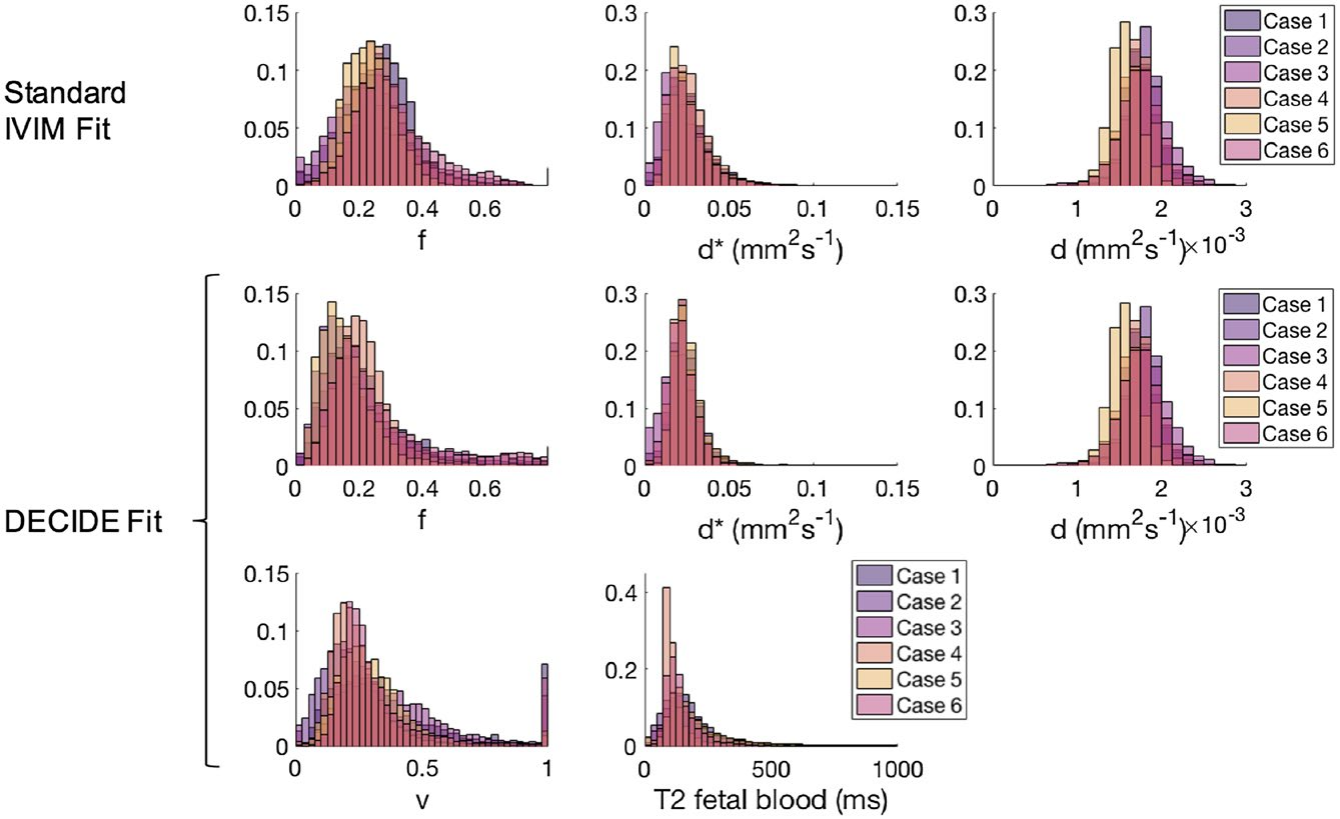
Voxel‐wise whole‐placenta parameter histograms for the 6 placenta data sets. Row 1 shows f, d*, and d for the standard IVIM fit. Rows 2 and 3 show f, d*, d, v, and T2 relaxation time of fetal blood for the DECIDE fit (Equation ((2)))

Placenta parameter maps of example slices for 1 example case is shown in Figure 4 (parametric maps for all subjects are shown in Supporting Information Figure S2). f and v show a heterogeneous, lobulated pattern. Figure 5 shows histograms of the voxel‐wise fit for each placenta case for each parameter, using the standard IVIM model and the DECIDE model. Table 2 shows a comparison of the mean (±standard deviation) for each parameter for the standard IVIM fit and the DECIDE fit for the placenta. f is slightly lower using the DECIDE fit than the standard IVIM fit.^16^ There was a high v, showing a large third compartment within the normal placenta. Mean T2 relaxation time of the fetal blood component was 144.89 (±54.42) ms.

### 3.4 Maternal‐fetal blood volume ratio

Fetoplacental perfusion is believed to be regulated to match maternal perfusion, through regulation of stem artery vasoconstriction.^35^ It is therefore interesting to be able to compare maternal‐to‐fetal blood volume fraction throughout the placenta. We do this by estimating the corrected volume ratio: (1-(f)) /f, where the correction of v takes into account the formulation used in Equation ((2)), which estimates v from the fraction of non‐IVIM signal.

**Figure 6:**
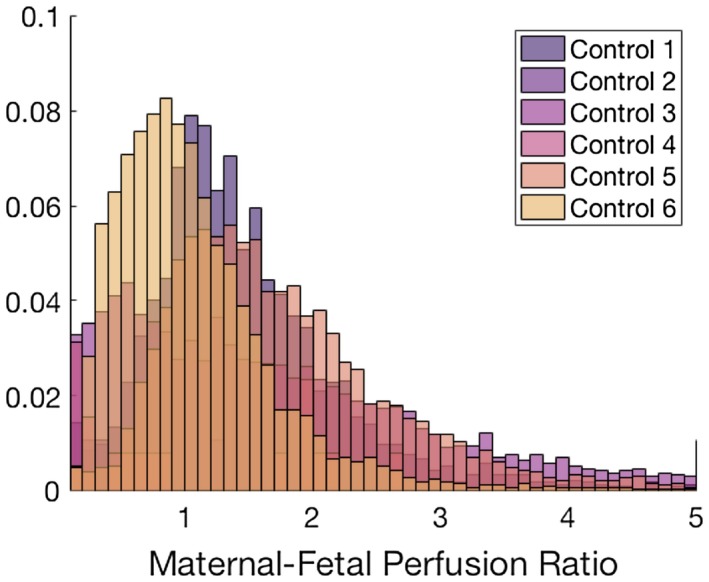
Histograms of voxel‐wise fit of the maternal‐fetal blood volume ratio for the 6 placenta data sets

A histogram of the voxel‐wise fit of this maternal‐fetal blood volume ratio in each case is shown in Figure 6. The cases show similar distributions, with the majority of voxels showing a ratio between 0.5 and 1.5 in all cases. The mean ratio for the 6 control cases was 1.16 (±0.6).

### 3.5 Fetal blood oxygen saturation

**Figure 7:**
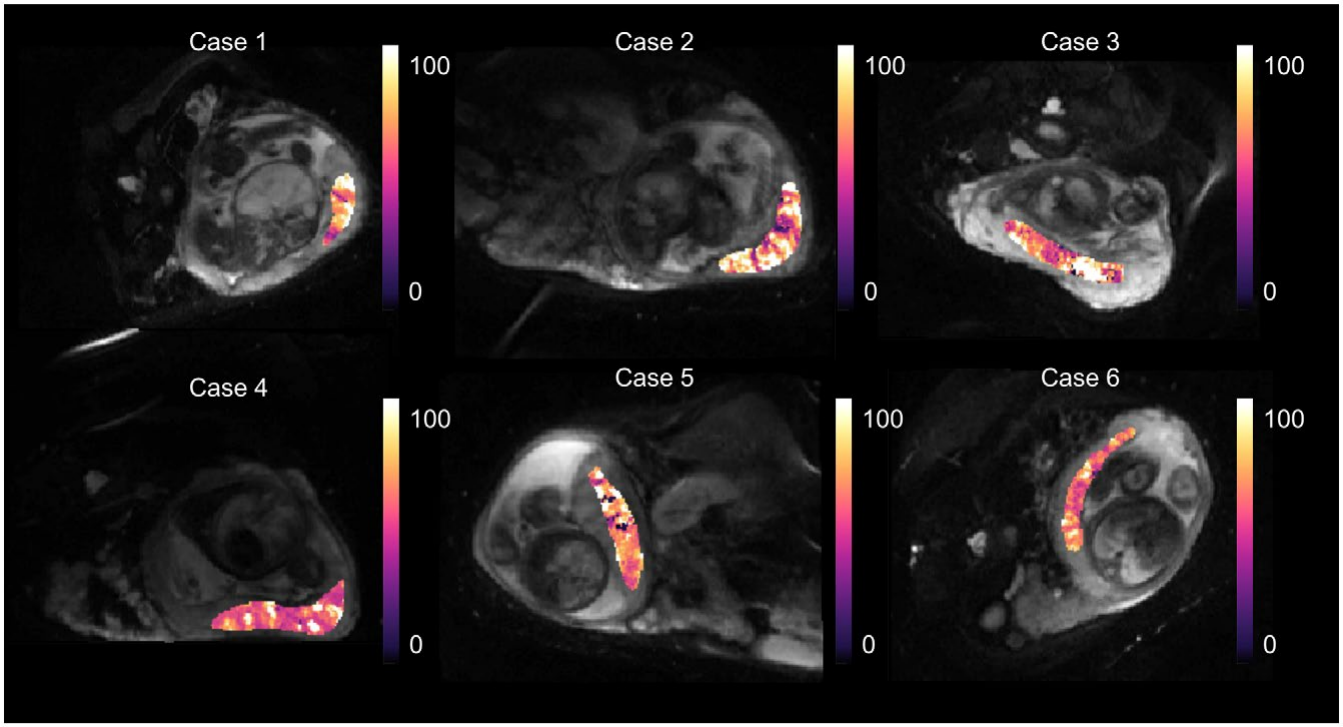
Parametric maps for the voxel‐wise fit of fetal blood oxygen saturation, derived from fetal blood T2 relaxation time. A histogram showing the voxel‐wise fit for the whole‐placenta data is shown in Figure 8. Mean fetal blood saturation over the whole data set (s < 100%) was 72.93 (±20.11)%

Voxel‐wise estimates of fetal blood oxygen saturation were calculated for the whole placenta in the 6 cases using Equation ((3)). Example slice parametric maps are shown for every case in Figure 7. These show a lobulated appearance, suggestive of heterogeneity throughput the placental tissue.^11^, ^12^

**Figure 8:**
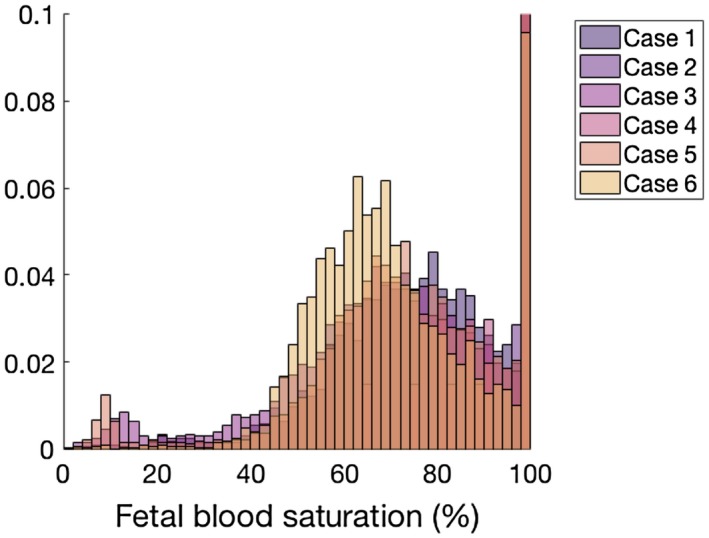
Histogram showing the voxel‐wise fit of fetal blood oxygen saturation over the whole placenta for the 6 cases (Equation ((3))). There are few voxels with saturation less than 45%. Mean fetal blood saturation (s < 100%) over the whole data set was 72.93 (±20.11)%

A histogram showing the voxel‐wise fit for the whole placenta data is shown in Figure 8. Mean fetal blood saturation over the whole data set (s < 100%) was 72.93 (±20.11)%.

## 4 DISCUSSION

We have developed a new technique for the analysis of structure and function of the placenta using multiple MRI contrast techniques and reported results in 6 singleton control placentae. The multimodal data presented in this work and the associated model have allowed an interpretation of the diffusion and relaxometry properties in terms of the physiological compartments of the placental tissue. We showed comparable results using our technique to previous studies, where measuring T2 simultaneously with IVIM has been shown to reduce f compared to isolated DWI.^16^ The T2 of both myometrial tissue and maternal blood have been previously estimated, and we found comparable values in our work.^32^, ^34^ We then applied our technique to placental tissue, simultaneously estimating the novel maternal/fetal blood volume ratio and fetal blood saturation. These parameters allow us to investigate the placental tissue in new detail, and the measurements that we obtain were in good agreement with known estimates from invasive fetal blood saturation measurement.^33^

The myometrium is important because it allows investigation of maternal perfusion without the interference of fetal perfusion signal and may thus be a useful tissue to investigate in its own right. The myometrial tissue is also useful for estimation of maternal blood T2 relaxation time given that this parameter may be fixed within the full DECIDE model. Placental insufficiency is caused by poor spiral artery remodeling in the first trimester, leading to low‐volume, high‐pressure blood flow. It is therefore possible that myometrial tissue properties may be an early marker of poor invasion and high risk of placental insufficiency. Both the standard IVIM and myometrial model fitting gave high values for f and d* in this normal cohort, which is expected within the highly vascular pregnant retroplacental myometrium.

In the placenta, the DECIDE model gave a lower f value than the standard IVIM model, as observed in similar work.^16^ f and d* values, however, remain high, in keeping with this biomarker representing fetal perfusion of the placenta. There was also a significant v fraction, which is expected given that this is thought to represent maternal placental perfusion and agrees with our expectation that this is a relevant physiological parameter in this unique tissue. Flow matching was assessed as the ratio of maternal‐to‐fetal blood volume. Flow matching is a possible consequence of the regulation of stem artery vasoconstriction, which has been proposed as a mechanism for control of perfusion in response to local oxygenation conditions.^35^ The distribution we observed had a relatively broad distribution of values, but it is possible that changes to this distribution associated with pathology may represent a new biomarker of disruption to placental flow matching in pathology.

Fetal blood T2 relaxation time estimated by DECIDE was lower than the value attributed to blood T2 in the adult. Fetal hemoglobin functions differently from adult hemoglobin because it has a greater oxygen‐binding affinity than adult hemoglobin, allowing the developing fetus to take oxygen from maternal blood more efficiently. However, fetal blood also has a higher hematocrit than adult blood and lower oxygen saturation. At 30 weeks gestational age, deoxygenated blood in the umbilical artery is estimated to be 65% saturated, and oxygenated blood in the umbilical vein is estimated to be 85% saturated.^33^ Hematocrit and oxygen saturation are known to affect T2 relaxation times, with T2 time decreasing as hematocrit increases and oxygen saturation decreases.^27^, ^34^ In addition, differences in hemoglobin concentration will have significant impact on the relationship between SpO2 and T2 that are not currently included in our model. It is therefore feasible for the T2 of fetal blood to be significantly lower than the T2 of maternal blood. We have presented a new way to estimate fetal blood oxygenation without the requirement for an invasive procedure.

Technically, there are a number of limitations of the methodology used in this work. The work is carried out at 1.5T with a fixed b‐value step size of 50 $s.mm^{-2}$. The DECIDE model is itself a simplification of the physiological processes and MR physics of the placenta. To help with validation of the measurements we have obtained, future work will incorporate measurements of directionality as in a previous work^19^ or be combined with measurements of T2* related to oxygenation.^11^, ^22^ Separation of the T2 signal into 2 compartments is likely to mask a spectrum of T2 compartments across a wide range and our use of literature values of T2 is a possible source of bias. Similarly, the perfusive and diffusive properties of the fetal and maternal circulations almost certainly overlap, particularly for high‐velocity perfusion close to the exit of the spiral arteries,^6^ and further work is needed to investigate how these signals may be fully disentangled, for instance by using ASL.^14^, ^15^ Flow in fetal capillaries is generally around 1 mm/s, whereas the flow velocity of the maternal blood in the intervillous space may show a significant spatial gradient and may overlap significantly across this range, particularly if there is variation in the degree of spiral artery remodeling. A further limitation of this work is the difficulty in registering data. Although the placenta is easier to image and register than the moving fetus, it is still challenging, being an intra‐abdominal organ, and therefore subject to nonrigid motion from maternal breathing and bowel motion. In addition, large fetal movements can distort the uterus, or compress the placenta unpredictably, making registration more challenging than other organs affected by respiratory motion.

## 5 CONCLUSION

In summary, we have developed a novel MRI technique that can be used to reveal new features of placental physiology by making use of combined relaxometry and diffusion‐weighted MRI. The novel acquisition of combined diffusion and multiecho T2 imaging within clinically feasible time frames allows us to generate novel predictive measurements of clinically and histologically relevant physical parameters, such as the maternal‐to‐fetal flow matching conditions and fetal blood oxygen saturation. In the placenta, this may help us close the gap between the features we observe during imaging and the underlying physiological properties of the tissue that can be observed postpartum, physiological properties that we may in the future be able to modify with fetal therapy.

## Supporting information

FIGURE S1 Parametric maps for the T2‐IVIM fit for one slice from the 4 cases where myometrial analysis was feasible. Rows show cases, columns show parameter maps, from left to right d*, d, T2 maternal blood and T2 myometrium
FIGURE S2 Parametric maps for the DECIDE fit (Eq. 2) for the 6 control singleton pregnancies. Rows show cases, columns show parameter maps, from left to right, f, d*, d, v, and T2 fetal blood
