# Supplementary material for: Separating fetal and maternal placenta circulations using multiparametric MRI
Source: Magn Reson Med. 2018 Sep 21;81(1):350–61. doi: 10.1002/mrm.27406 (PMC6282748; doi:10.1002/mrm.27406)
Supplement: Supplementary file 1 — FIGURE S1 Parametric maps for the T2‐IVIM fit for one slice from the 4 cases where myometrial analysis was feasible. Rows show cases, columns show parameter maps, from left to right d*, d, T2 maternal blood and T2 myometrium FIGURE S2 Parametric maps for the DECIDE fit (Eq. 2) for the 6 control singleton pregnancies. Rows show cases, columns show parameter maps, from left to right, f, d*, d, v, and T2 fetal blood [file MRM-81-350-s001.docx]

Separating fetal and maternal placenta circulations using multi-parametric MRI

Supporting Information

##

***
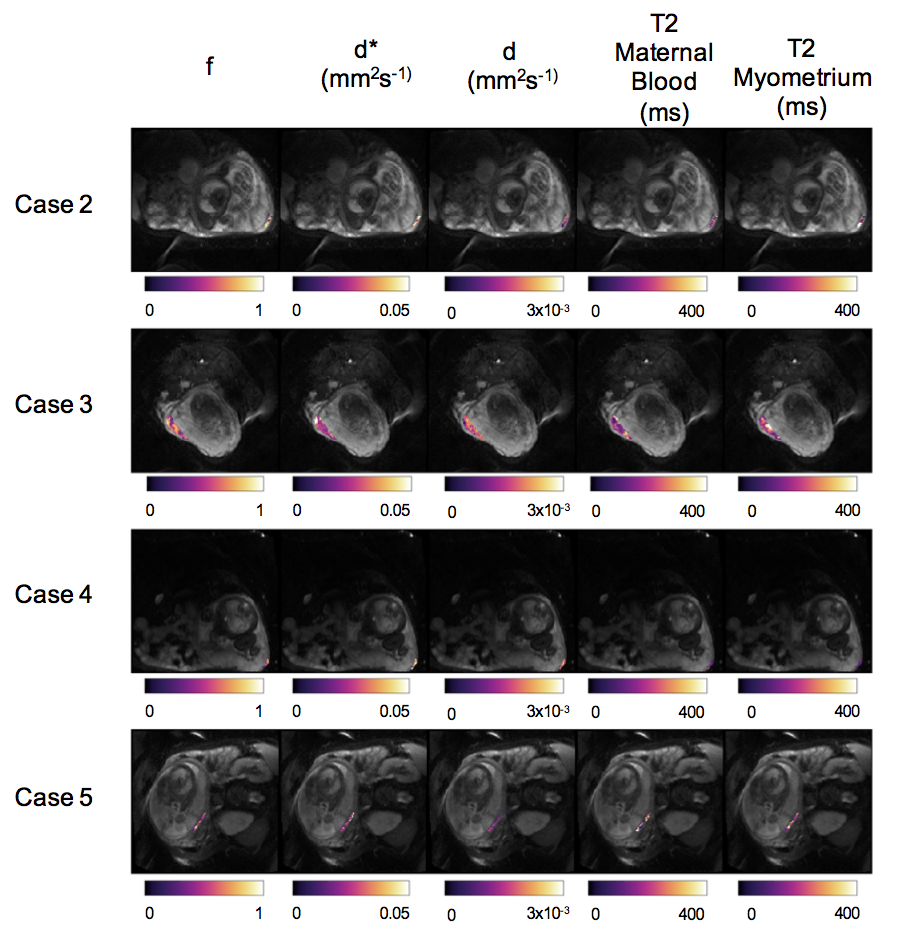
***

**Figure S1** Parametric maps for the T2-IVIM fit for one slice from the 4 cases where myometrial analysis was feasible. Rows show cases, columns show parameter maps, from left to right, $f$, d*, d, T2 maternal blood and T2 myometrium.

***
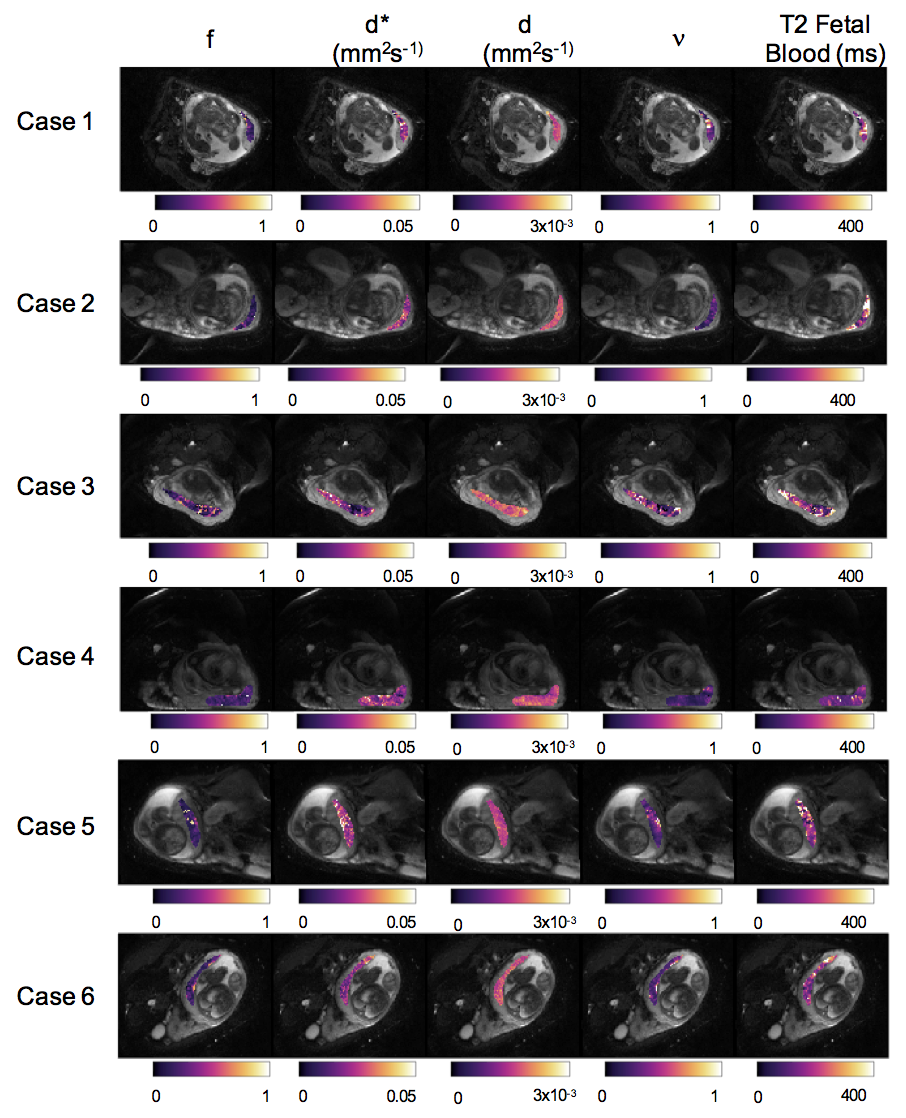
***

**Figure S2** Parametric maps for the DECIDE fit (Eq. 2) for the 6 control singleton pregnancies. Rows show cases, columns show parameter maps, from left to right, $f$, d*, d, ν, and T2 fetal blood.
